# Stepwise asynchronous telehealth assessment of patients with suspected axial spondyloarthritis: results from a pilot study

**DOI:** 10.1007/s00296-023-05360-z

**Published:** 2023-06-14

**Authors:** Labinsky Hannah, Rohr von Sophie, Raimondo Maria Gabriella, Bohr Daniela, Morf Harriet, Horstmann Britta, Seese Felix, Proft Fabian, Muehlensiepen Felix, Boy Katharina, Kuhn Sebastian, Schmalzing Marc, Vuillerme Nicolas, Schett Georg, Ramming Andreas, Knitza Johannes

**Affiliations:** 1 Department of Internal Medicine 3, Rheumatology and Immunology Friedrich, Alexander University Erlangen-Nürnberg and Universitätsklinikum Erlangen, Ulmenweg 18, 91054 Erlangen, Germany; 2 Deutsches Zentrum für Immuntherapie, Friedrich-Alexander University Erlangen-Nürnberg and Universitätsklinikum Erlangen, Erlangen, Germany; 3 Department of Internal Medicine 2, Rheumatology/Clinical Immunology, University Hospital Würzburg, Oberdürrbacher Straße 6, 97080 Würzburg, Germany; 4 Department of Gastroenterology, Infectiology and Rheumatology (Including Nutrition Medicine), Charité Universitätsmedizin Berlin, Berlin, Germany; 5 Brandenburg Medical School, Centre for Health Services Research Brandenburg, Rüdersdorf, Germany; 6 Brandenburg Medical School, Faculty of Health Sciences Brandenburg, Neuruppin, Germany; 7 Université Grenoble Alpes, AGEIS, Grenoble, France; 8 Institute of Digital Medicine, Philipps-University & University Hospital of Giessen and Marburg, Marburg, Germany; 9 Institut Universitaire de France, Paris, France; 10 LabCom Telecom4Health, Orange Labs & University Grenoble Alpes, CNRS, Inria, Grenoble INP-UGA, Grenoble, France

**Keywords:** Symptom checker, Telemedicine, Telehealth, Diagnosis, Spondyloarthritis, Health service research

## Abstract

Patients with axial spondyloarthritis (axSpA) suffer from one of the longest diagnostic delays among all rheumatic diseases. Telemedicine (TM) may reduce this diagnostic delay by providing easy access to care. Diagnostic rheumatology telehealth studies are scarce and largely limited to traditional synchronous approaches such as resource-intensive video and telephone consultations. The aim of this study was to investigate a stepwise asynchronous telemedicine-based diagnostic approach in patients with suspected axSpA. Patients with suspected axSpA completed a fully automated digital symptom assessment using two symptom checkers (SC) (bechterew-check and Ada). Secondly, a hybrid stepwise asynchronous TM approach was investigated. Three physicians and two medical students were given sequential access to SC symptom reports, laboratory and imaging results. After each step, participants had to state if axSpA was present or not (yes/no) and had to rate their perceived decision confidence. Results were compared to the final diagnosis of the treating rheumatologist. 17 (47.2%) of 36 included patients were diagnosed with axSpA. Diagnostic accuracy of bechterew-check, Ada, TM students and TM physicians was 47.2%, 58.3%, 76.4% and 88.9% respectively. Access to imaging results significantly increased sensitivity of TM-physicians (*p* < 0.05). Mean diagnostic confidence of false axSpA classification was not significantly lower compared to correct axSpA classification for both students and physicians. This study underpins the potential of asynchronous physician-based telemedicine for patients with suspected axSpA. Similarly, the results highlight the need for sufficient information, especially imaging results to ensure a correct diagnosis. Further studies are needed to investigate other rheumatic diseases and telediagnostic approaches.

**Supplementary Information:**

The online version contains supplementary material available at 10.1007/s00296-023-05360-z.

## Introduction

Axial spondyloarthritis (axSpA) is a common inflammatory rheumatic disease with an estimated prevalence of 0.3–1.4% worldwide [1–3]. The diagnostic delay of axSpA patients remains a major challenge, remaining unacceptably long with around 7 years in Europe [4, 5]. Untreated disease deteriorates prognosis, decreases quality [6] of life and leads to functional disability and economic losses [7, 8]. The increasing shortage of rheumatologists and the simultaneous rise in demand are likely to increase the diagnostic delay even further [9].

The European Alliance of Associations for Rheumatology (EULAR) recently highlighted the growing importance of telehealth for rheumatology [10], however also demonstrated the scarcity of evidence [11]. In the underlying systematic review [11], the authors identified only two published studies [12, 13] regarding remote diagnosis. These two studies investigated traditional resource intensive telemedicine strategies, where patients with different rheumatic diseases and additional health care professionals (HCP) communicated synchronously with rheumatologists. Encouragingly, both studies demonstrated high diagnostic accuracy and patient acceptance. Asynchronous telemedicine has been gaining popularity, in particular due to the increased flexibility and lower need of human resources. Symptom checkers (SC) are an extreme example of asynchronous telehealth, as they attempt to detect a disease only based on medical history and without any HCP review of data. Compared to traditional face-to-face diagnosis, SC showed low diagnostic accuracy for several rheumatic conditions, [14–16] including axSpA [17]. Only when rheumatologists were limited to medical history only, SC showed a significantly higher accuracy [18]. Similarly, Ehrenstein et al. demonstrated that even experienced rheumatologists needed additional imaging and laboratory data to reach a satisfactory diagnostic accuracy [19]. We hypothesized that an accurate asynchronous telehealth diagnosis of patients with suspected axSpA is possible, if physicians only had access to enough information, including medical history, laboratory parameters and imaging. Thus, our study investigated an asynchronous telediagnostic approach in patients with suspected axSpA.

## Methods

Newly referred adult patients with suspected axSpA were included in this study. Exclusion criteria were a known diagnosis, a previous rheumatologist appointment and unwillingness or inability to comply with the protocol. This prospective study was approved by the institutional review board (IRB) of the Medical Faculty of the University of Erlangen-Nürnberg (21–357-B) and conducted in compliance with the Declaration of Helsinki. All study patients provided written informed consent prior to study participation.

### Symptom checker diagnosis

Prior to their rheumatology visit, patients completed two SC, bechterew-check (BC; www.bechterew-check.de) and Ada (www.ada.com). BC is an axSpA-specific online questionnaire based on the ASAS criteria, consisting of 16 questions, classifying answers as likely or unlikely for axSpA. Ada is a freely-available medical app not limited to rheumatology. The artificial intelligence-driven chatbot questions are dynamically chosen, and the total number varies depending on the previous answers given. Ada provides a top (D1) and up to five disease suggestions (D5), their respective probability and urgency advice. Disease suggestions were compared to the final diagnosis reported on the discharge summary report. Patient acceptance of symptom checkers was measured using the net promoter score [20] (NPS), which is based on a 11-point numeric rating scale (0–10). Answers between 0 and 6 are categorized as detractors, 7–8 as passives and 9–10 as promoters. The NPS is equal to the percentage of promoters subtracting the percentage of detractors.

### Healthcare professional-based telehealth diagnosis supported by symptom checkers

After the patient visit, two independent medical students (4 and 5 years of completed studies, respectively) who received a brief presentation of axSpA diagnosis (15 min) and three physicians (1 resident, 2 board-certified rheumatologists) were consecutively presented (1) symptom checker summaries from both BC and Ada, (2) CRP and HLA B-27 results (venous; gold standard) and (3) radiology reports. After each step, participants had to state if axSpA was present or not (yes/no), rate their perceived diagnostic confidence on an 11-point numeric rating scale (NRS 0–10) and record diagnostic step completion time in seconds. Disease suggestions were compared to the final diagnosis reported on the discharge summary report.

### Statistical analysis

Due to the exploratory character of the trial, no formal sample size calculation was performed. Following recommendations for pilot studies [21], the number of patients was set at 40. Statistical analysis was performed using Microsoft Excel 2019 and GraphPad Prism 8. The P value is reported and P values less than 0.05 were considered significant. Additionally, for nominal variables, the 95% CI of the difference between medians is reported and for categorical variables, the 95% CI and Odd’s ratio are indicated. Patient-to-patient comparisons were summarized by median and interquartile range *(IQR, interquartile range 25*^*th*^* and 75*^*th*^* percentiles) for interval data and as absolute (n) and relative frequency (percent) for nominal data*. Statistical differences were assessed by Mann–Whitney-*U* test and Kruskal–Wallis test with Dunn’s test for multiple comparisons and Fisher's Exact Test for categorical variables. Results were reported following the STAndards for the Reporting of Diagnostic accuracy studies guideline [22]. Diagnostic accuracy was evaluated referring to sensitivity, specificity and overall accuracy. Asynchronous TM-based sensitivity and specificity were statistically compared after each diagnostic step using McNemar’s test.

## Results

Baseline patient characteristics are shown in Table 1. 17/36 (47.2%) of patients were diagnosed with axSpA. There were three study dropouts due to missed appointments and one patient refused to participate. Median age was 37.2 years, 21/36 (58.3%) were female. All patients had lower back pain for more than 3 months.

**Table 1:** Patient characteristics

Patient characteristics	All patients (*n* = 36)	axSpA (*n* = 17)	No. axSpA (n = 19)	*P* value	95% CI (and Odd’s ratio)*
Age, Mdn (IQR)	37.19 (20.6)	34.4 (15.2)	39.9 (18.0)	0.24	– 3.6 to 12
BMI, Mdn (IQR)	24.6 (5.1)	25.8 (2.3)	24.4 (8.2)	0.59	– 3.4 to 2.5
Active smoker status, *N* (%)	5 (13.9)	4 (21.1)	1 (5.9)	0.16	0.7 to 70.9 (5.5)
Female gender, *N* (%)	21 (58.3)	7 (41.2)	14 (73.7)	0.09	0.1 to 1.0 (0.3)
Chronic lower back pain, *N* (%)	36 (100)	17 (100)	19 (100)	1.00	–
Peripheral enthesiopathy, *N* (%)	10 (27.8)	6 (35.3)	4 (21.1)	0.46	0.5 to 7.5 (2.0)
Peripheral arthralgia	15 (41.7)	6 (35.3)	9 (47.4)	0.52	0.2 to 2.4 (0.6)
Elevated baseline CRP, *N* (%)	7 (38.9)	4 (23.5)	3 (15.8)	0.68	0.4 to 7.3 (1.6)
HLA-B27 positive, *N* (%)	18 (50)	10 (58.8)	8 (42.1)	0.51	0.6 to 6.5 (2.0)
History of uveitis, *N* (%)	0 (0)	0 (0)	0 (0)	1.00	–
History of IBD, *N* (%)	0 (0)	0 (0)	0 (0)	1.00	–
History of psoriasis, *N* (%)	1 (2.8)	1 (5.9)	0 (0)	0.48	0.1 to Inf (Inf)
NSAR response, *N* (%)	27 (75)	15 (88.2)	12 (63.2)	0.13	0.9 to 23.0 (4.4)
Familiy history of axSpA	7 (38.9)	1 (5.9)	6 (31.6)	0.09	0.01 to 1.1 (0.14)
Family history of IBD	3 (8.3)	0 (0)	3 (15.8)	0.23	0.0 to 1.2 (0.0)
Family history of psoriasis	4 (11.1)	1 (5.9)	3 (15.8)	0.61	0.0 to 2.5 (0.3)
Baseline PtGA, Mdn (IQR)	5 (3)	4 (3)	5 (3)	0.36	– 2.0 to 1.0
Baseline Morning stiffness at T-1, Mdn (IQR)	30 (51.3)	30 (50)	30 (37.5)	0.86	– 27.0 to 20.0
Baseline BASDAI, Mdn (IQR)	4.2 (2.7)	4 (2.3)	4.3 (2.7)	0.47	– 1.0 to 2.0
BASMI > 0, *N* (%)	13 (36.1)	6 (35.3)	7 (36.8)	1.00	0.2 to 3.4 (0.8)

*Mdn* Median, *IQR* interquartile range, *BMI* body mass index, *IBD* inflammatory bowel disease, *VAS* visual analogue scale, *BASDAI* Bath Ankylosing Spondylitis Disease Activity Index, *BASFI* Bath Ankylosing Spondylitis Functional Index, *CI* Confidence interval, *Inf* infinity

Statistical significances between the axSpA and non-axSpA patients were determined by Mann Whitney *U* test for nominal variables and Fisher’s exact test for categorical variables

*For nominal variables 95, % CI of the difference between medians is reported. For categorical variables, 95, % CI and Odd’s ratio in parantheses are indicated

**Fig. 1:**
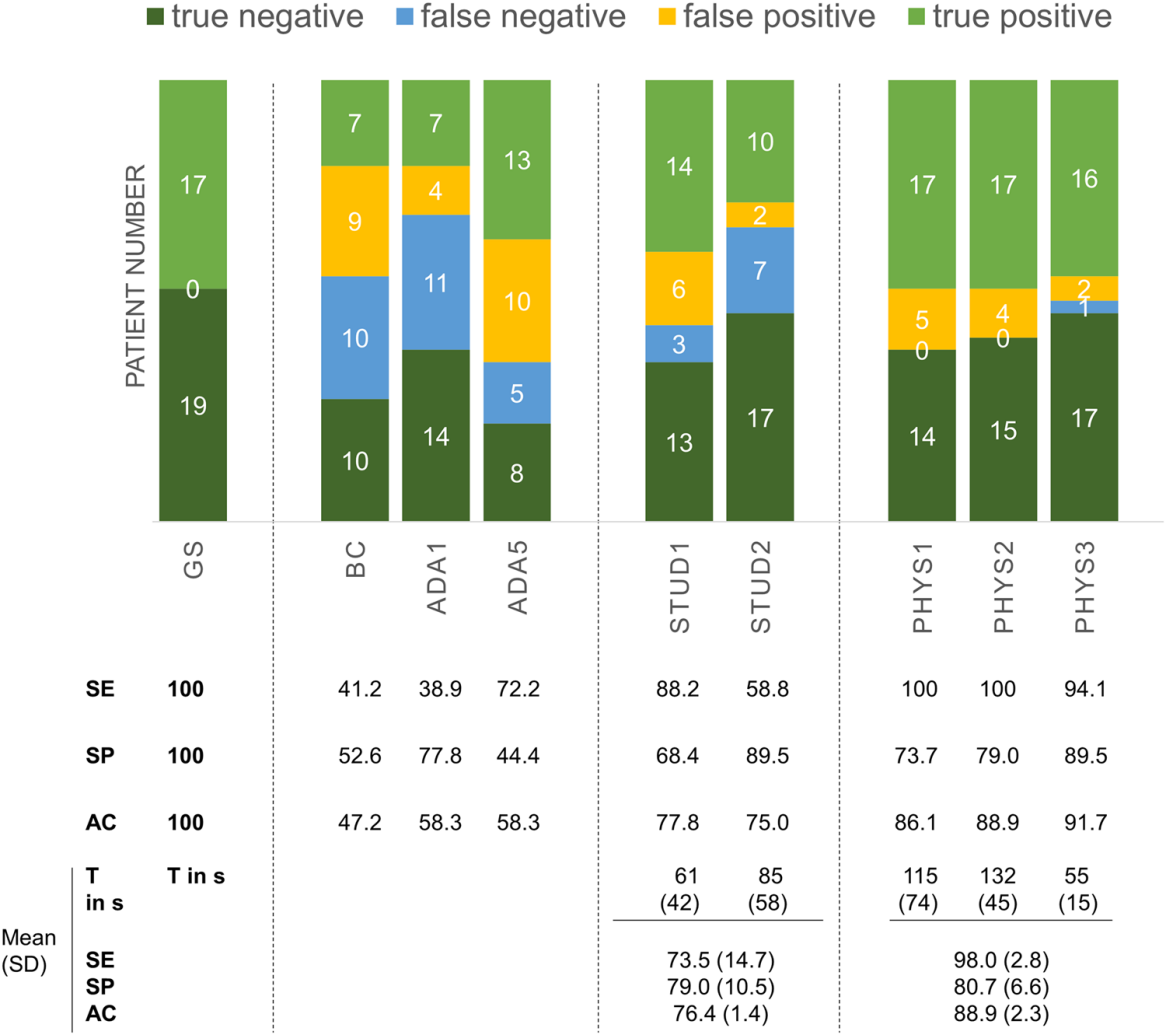
Diagnostic accuracy measures of symptom checkers (SC), students and physicians. Final diagnosis reported on the discharge summary report served as the gold standard (GS). Based on this, the sensitivity, specificity and diagnostic accuracy of the two SC bechterew-check (BC) and Ada (ADA1 = top1 diagnosis, ADA5 = top5 diagnoses) were determined. Students 1 + 2 (STUD1 + 2) and physician 1–3 (PHYS1-3) decided asynchronously based on SC results, results for CRP and HLA-B27 and imaging, without ever having actually seen the patient. The mean time (T) for telehealth diagnosis per patient case vignette is listed in seconds (s). Mean diagnostic accuracy values are listed in the three lower rows of the table. *SE* sensitivity, *SP* specificity, *AC* accuracy

The diagnostic accuracy of both SC (BC: 47.2%; ADA: 58.3%) was inferior to medical students and physicians (Fig. 1). Diagnostic accuracy increased with increasing information for both students and physicians (see online supplemental material S1). Giving physicians and medical students only access to SC reports resulted in a mean diagnostic accuracy of 54.2 ± 4.2 (BC) vs. 62.5 ± 4.2 (ADA) and 55.2 ± 3.7 (BC) vs. 58.5 ± 0.2 (Ada), respectively.

**Fig. 2:**
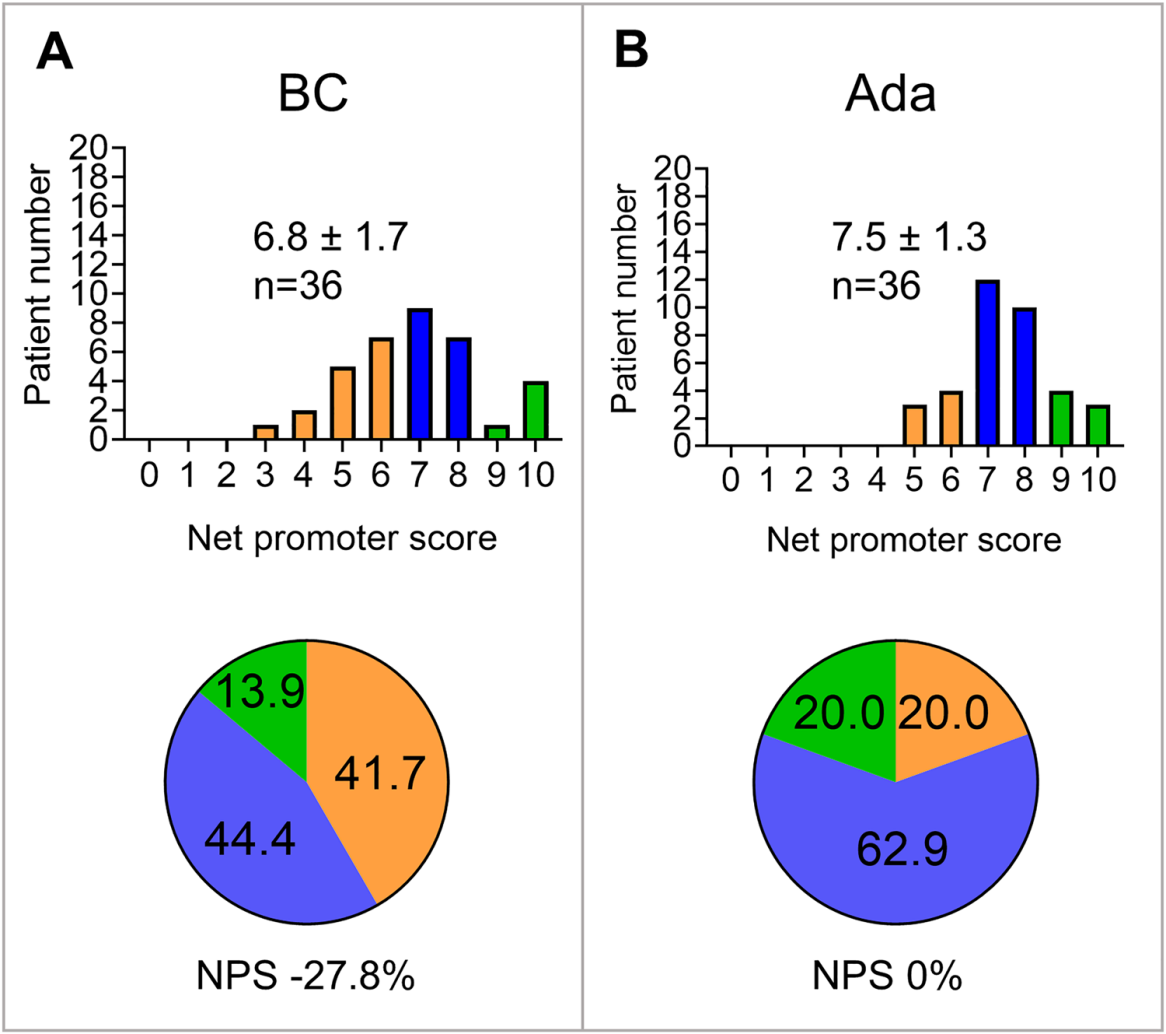
Patient acceptance for symptom checkers is displayed as bar graphs and pie charts. The proportion of detractors (0–6) is shown in orange, the proportion of neutrals (7–8) in blue and the proportion of promoters (9–10) in green

With access to all diagnostic information including SC reports, CRP and HLA-B27 results and imaging, students’ telehealth diagnostic accuracy still appeared limited (76.4 ± 1.4%), results of the three tele-rheumatologists showed a high mean sensitivity (98.0 ± 2.8%) and overall diagnostic accuracy (88.9 ± 2.3%) (Fig. 1). Interestingly, median diagnostic confidence of false axSpA classification was not significantly lower compared to correct axSpA classification for both students and physicians (online supplemental material S2). Similarly, the reported diagnostic probability of Ada did not significantly differ between correct diagnosis and false diagnosis (median diagnostic probability 0.4 vs. 0.5, 95% CI of difference -0.1 to 0.2; p = 0.46), see online supplemental material S3. Imaging significantly increased the sensitivity of the three individual telehealth physicians and one individual student (*p* < 0.05 by McNemar’s test, median physician’s sensitivity 64.71% w/o imaging vs. 100% including imaging, 95% CI of difference 22.7% to 52.9%), see online supplemental material S1. Mean time for asynchronous telehealth diagnosis varied between 55 and 132 s (Fig. 1). Patient acceptance of symptom checkers was poor with NPS ratings of 0% for Ada (mean ± SD 7.5 ± 1.3) and -27.8% for BC (6.8 ± 1.7), see Fig. 2.

## Discussion

In this cross-sectional study of patients with suspected axSpA, we demonstrated the feasibility of asynchronous telediagnosis for the majority of patients. Asynchronous telehealth physicians reached a mean diagnostic accuracy of 88.9% and sensitivity of 98.0% and needed an average of only 1–2 min per case. Previous remote rheumatology diagnosis studies used a resource intensive synchronous video consultation approach involving additional personnel (junior doctor, nurse, general practitioner) reporting accuracies of 40% [23], 79% [13] and 97% [12]. In our study, the significant increase in sensitivity by gaining access to imaging data highlights the importance of having access to all crucial information, confirming a study by Ehrenstein et al. [19], that also examined relative contributions of sequential diagnostic steps. Being limited to medical history data only, such as symptom checkers are, it has been shown previously [19] that also experienced rheumatologists only reach a very limited diagnostic accuracy of 27%. The low diagnostic accuracy of symptom checkers in this study is similar to previous studies [16, 24]. Machine learning could however improve diagnostic accuracy [25]. As expected, in a recent large video consultation diagnostic study, accuracy was very high (100%) in disciplines that were mainly based on imaging and laboratory data compared to disciplines that heavily rely on physical examination [26]. The limitations of the physical examination in video consultations restrict remote diagnostic accuracy [23]. Increasing availability of professional imaging, and new smartphone-based techniques [27] slowly reduce these restrictions. However, substantial prevalence of inflammatory MRI lesions among healthy individuals [28] warrant for careful consideration. We previously reported high accuracy and patient acceptance regarding at-home capillary self-sampling for CRP and antibody analysis [29–31] to support telehealth diagnosis and monitoring. Importantly, the diagnostic uncertainty is only partly perceived by physicians, as can be seen from the low difference in perceived diagnostic confidence for false and correct diagnoses in this trial and a previous one [18]. Therefore in clinical routine this diagnostic approach should currently rather be used to triage patients but not prevent actual on-site visits.

To our knowledge, this is the first study investigating an asynchronous hybrid diagnostic telehealth approach in rheumatology. Despite its small size and its monocentric study nature, this study adds important evidence on telemedicine in rheumatology, as they were requested by EULAR [10]. Preliminary data of this study was presented at the American College of Rheumatology congress 2022 [32]. Our results have to be confirmed in larger studies in axSpA and can be rolled out to other diseases. Confirmation of cost-effectiveness will be crucial for wider implementation.

## Conclusion

In regard of the persistently long diagnostic delay of patients with axial spondyloarthritis new innovative strategies should be evaluated. This study underlines the potential of asynchronous physician-based telemedicine to diagnose patients with axSpA. Access to imaging results was crucial for a correct diagnosis. Further studies are needed to investigate other rheumatic diseases and different telediagnostic approaches.

### Supplementary Information

Below is the link to the electronic supplementary material.

Supplementary Supplemental material S1. Diagnostic accuracy measures of students and physicians. SE, sensitivity; SP, specificity; AC, diagnostic accuracy; SD standard deviance file1 (TIF 1067 KB)

Supplementary Supplemental material S2. Diagnostic confidence of telehealth physicians and students. Median diagnostic confidence of correct vs. low axSpA classification 8.0 vs. 7.5 (95% CI of the difference between medians -2.0-0; p=0.08) for physicians and 8.0 vs. 7.0 (95% CI of difference -2.0-0; p=0.11) for students file2 (TIF 300 KB)

Supplementary Supplemental material S3. Ada’s reported diagnostic probabilities file3 (TIF 206 KB)

## Data Availability

The raw data supporting the conclusions of this article will be made available by the authors, without undue reservation.
