# Supplementary figures and images for: Stepwise asynchronous telehealth assessment of patients with suspected axial spondyloarthritis: results from a pilot study
Source: Rheumatol Int. 2023 Jun 14;44(1):173–80. doi: 10.1007/s00296-023-05360-z (PMC10766678; doi:10.1007/s00296-023-05360-z)

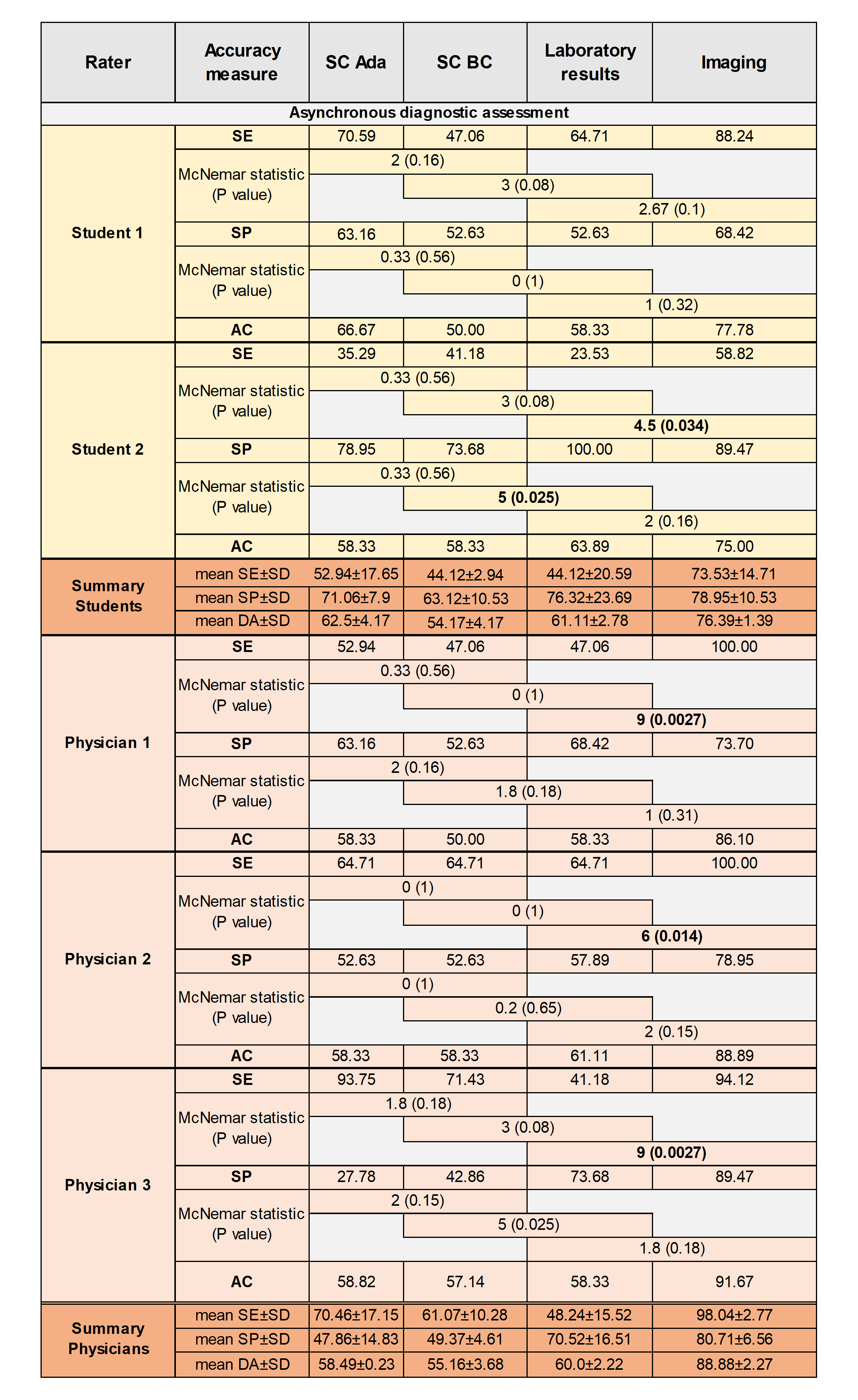

Supplement: Supplementary file 1 — Supplementary Supplemental material S1. Diagnostic accuracy measures of students and physicians. SE, sensitivity; SP, specificity; AC, diagnostic accuracy; SD standard deviance file1 (TIF 1067 KB) [file 296_2023_5360_MOESM1_ESM.tif]

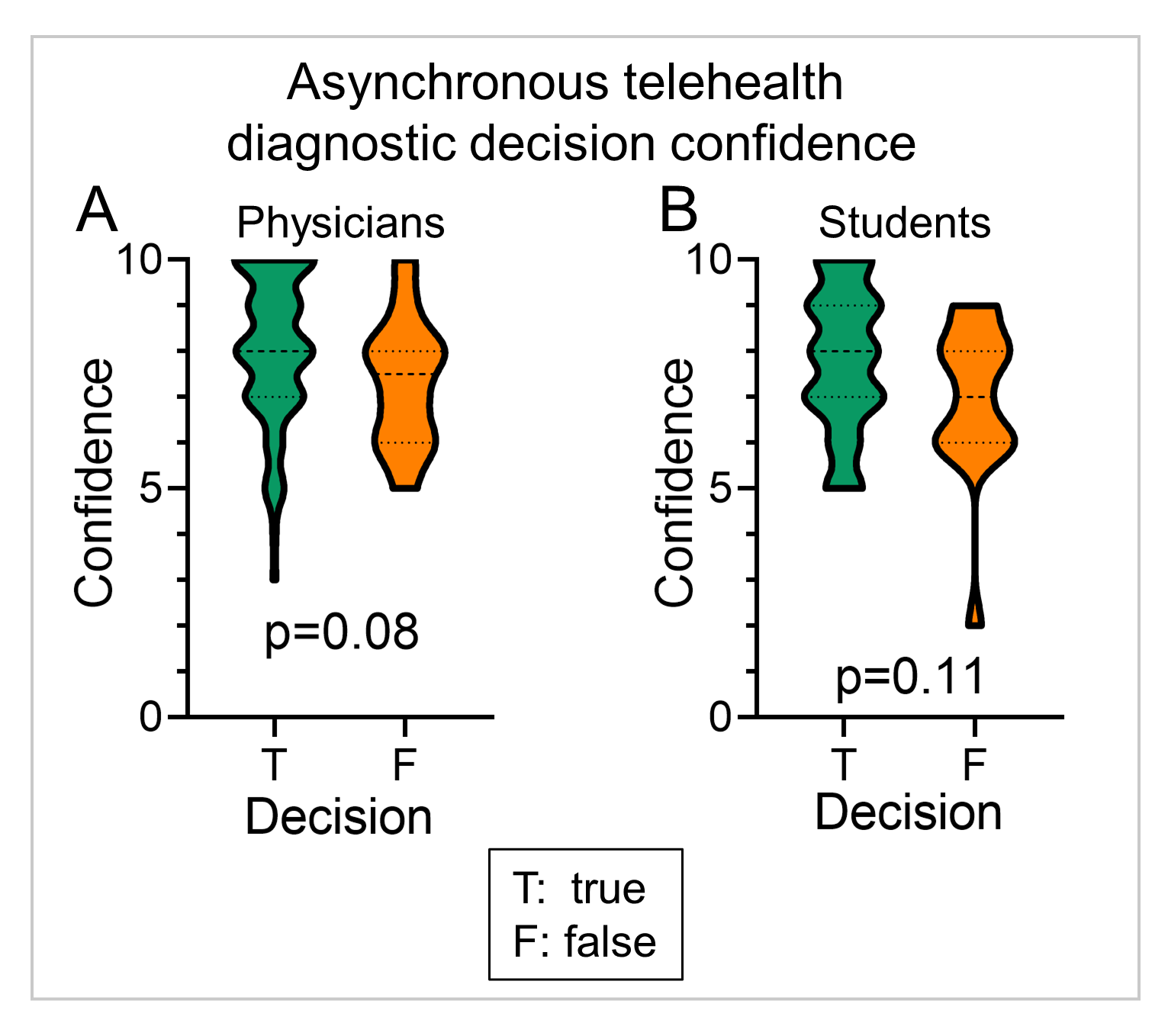

Supplement: Supplementary file 2 — Supplementary Supplemental material S2. Diagnostic confidence of telehealth physicians and students. Median diagnostic confidence of correct vs. low axSpA classification 8.0 vs. 7.5 (95% CI of the difference between medians -2.0-0; p=0.08) for physicians and 8.0 vs. 7.0 (95% CI of difference -2.0-0; p=0.11) for students file2 (TIF 300 KB) [file 296_2023_5360_MOESM2_ESM.tif]

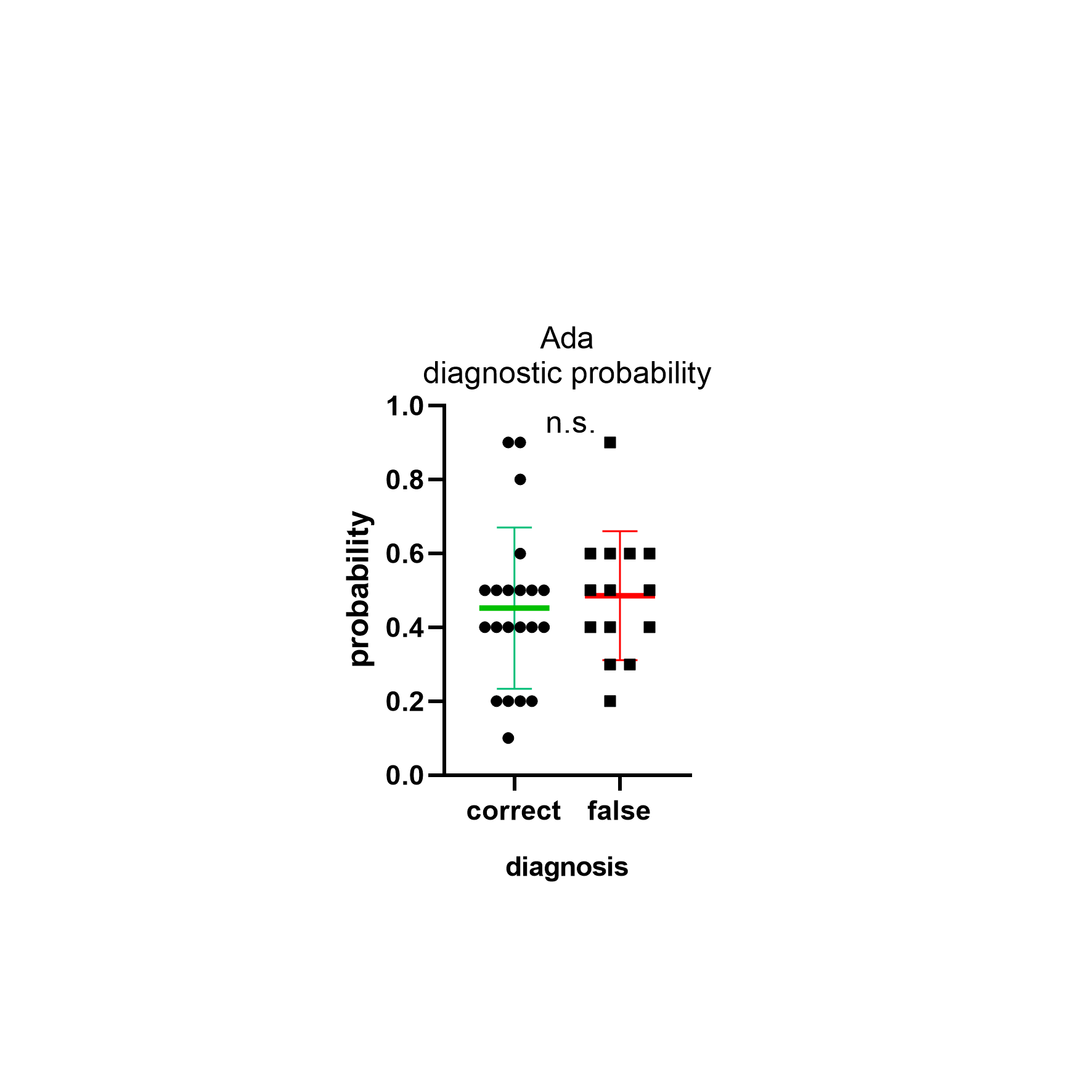

Supplement: Supplementary file 3 — Supplementary Supplemental material S3. Ada’s reported diagnostic probabilities file3 (TIF 206 KB) [file 296_2023_5360_MOESM3_ESM.tif]
